# Spasmodic Torticollis: Treatment by Resection of Cervical Nerves

**Published:** 1892-08-06

**Authors:** 


### SURGERY.

### SPASMODIC TORTICOLLIS TREATMENT BY RESECTION OF CERVICAL NERVES.

Several of the neuropathies which were formerly treated by nerve stretching, *i.e.*, if surgical measures were considered advisable at all, are now being treated by resection of the nerves. The treatment of spasmodic torticollis is so very unsatisfactory generally that we gladly welcome this procedure, which, as far as we can judge in these early days of the operation, is most promising. It is useless, we fear, to expect anything like an entirely satisfactory method of treatment until we know a little more about the pathology of this disease. Another fact which militates against the success of any single *method* of operation is the very marked variability of cases, different nerves and muscles being implicated in various cases observed. We believe that the treatment of spasmodic torticollis by excision of a portion of the spinal accessory nerve was first formulated by Mr. Campbell de Morgan in 1886, but the operation of division of the nerves supplying the rotatory muscles of the head was first devised and put into actual practice by Keen. His patient was a woman of fifty-four years of age. When first seen by her physician, Dr. Dercum, she stated that for two years she had suffered involuntary rotation of the head towards her left shoulder. The movement frequently recurred during conversation. The chin turned toward the left and was elevated slightly. She had distinct hypertrophy of the sterno-mastoid on the right side. She was operated upon by Dr. Ashhurst, who removed four inches of her spinal accessory nerve, both branches being embraced in the operation, and extreme traction being made upon the trunk. After the operation the spasms disappeared for a week, and then returned unchanged. Some time afterwards she was operated upon by Dr. Keen. The spasms ceased immediately after this second operation, but returned, though less violently, at the end of a week. A year later the patient's condition was markedly improved, although not cured.

Last year Mr. Petit reported twenty-six cases in which he had excised the spinal accessory nerve; and of these a good result was obtained in thirteen, seven were considerably benefited, two were only slightly improved, three were relieved temporarily only, and one died from erysipelas.

Noble Smith has recorded four cases, and in each case the relief from spasm has continued. He reports* that in the first instance the patient had had severe spasm for sixteen years. He removed a piece of the spinal accessory on the left side, and subsequently of the posterior branches of the second, third, and fourth cervical nerves, as well as every branch of nerve that he could find entering the splenius capitis muscle. This patient had remained free from spasm for two years, and may, therefore, be regarded as promising a permanent cure. Another case was that of a gentleman aged fifty-seven. He had spasmodic movements of the neck, head, and face. This affection had commenced in the right sterno-mastoid, and he was cured by excision of a piece of the right spinal accessory, and has remained well. More recently Mr. Smith has had two other cases. One was that of a lady aged forty-five, who suffered from spasmodic wryneck to the right, commencing fourteen years previous to operation. It had been most assiduously treated with drugs of all kinds, galvanism, and blisters to the spine. Nothing had given her relief. In this case the external rotators on the right side were acting more strongly than the left sterno-mastoid; therefore, he decided to operate upon the posterior branches of the cervical nerves in the first instance. Upon the first occasion he could not complete the operation, as the patient was showing signs of faintness under the anæsthetic. Six months later he operated upon the left spinal accessory nerve, removing half an inch of it. This removed the spasm of the sterno-mastoid. Subsequently Mr. Smith removed pieces from the second, third, and fourth posterior branches of the cervical nerves on the right side. This appeared to remove the spasms from the splenius, and what spasms were left to be confined to the deep rotators supplied by the suboccipital nerve which he had not operated upon. The patient, however, continued to improve further, and led the operator to hope that this patient will be cured without further operation. The fourth case was that of a lady over fifty, who had suffered from spasms for fifteen years. She had been under the care of Dr. Gervais, who had sent her to Mr. Smith, and who had come to the conclusion that any benefit to be obtained from drugs was only of a very transitory character, and that operation was the only treatment likely to do positive good. Excision of a piece of the left spinal accessory nerve relieved the patient from the spasms of that muscle. Other spasms were relieved, and Mr. Smith thinks that if necessary a further operation will produce a complete cure, and he promises a further report on this his fourth case.

Dr. Powers† has had a good result in a heavily-built man of thirty-seven years. He had exhibited a neuropathic tendency from boyhood, and his father was distinctly of a neurotic temperament. He had suffered from spasms for two years and a-half, at first slight in degree, but they gradually increased in severity and frequency till they were very marked. He had been given various drugs, electricity, and the like by several physicians, no measures being attended by permanent improvement. The head when left to itself was spasmodically rotated to the right to its fullest extent. These spasms were constant during the day, but were worse when the patient was fatigued, irritated, surprised, or among strangers. The spasmodic movements appeared to be a rotation of the atlas upon the axis. After very careful examination, his physician, Dr. Amidon, considered his affection to be confined to the posterior rotators, and recommended division or resection of the nerves supplying them. Dr. Powers reports that he had at the time read Dr. Keen's description of the operation Dr. Powers devised and carried out the operation described. A three-inch transverse incision was made at the back of the neck, beginning at the median line, an inch and a-quarter below the external occipital protuberance, and running forward. This was subsequently enlarged until it measured four inches and a-quarter in length. The parts divided were the trapezius and the posterior border of the splenius, until the complexus was reached and recognized, the trapezius being dissected up from it. After some difficulty the occipitalis major nerve was found at the upper part of the complexus, and outside of the intra-muscular aponeurosis of this muscle. Preserving the nerve, the complexus was divided transversely, after which the nerve was followed back to the posterior branch of the second cervical before that nerve gave off filaments to the obliquus inferior. The three muscles bounding the suboccipital triangle guided to the suboccipital nerve, which was followed back to its exit from the spinal canal. The external branch of the posterior division of the third curvical was found. Each nerve—*i.e.*, the first and second and third cervical, and the suboccipital—was followed well back to the spine, and a half to three quarters of an inch excised from each of the three.

On coming out of the operation, which lasted two hours, the patient had no spasms. Nearly a year later the patient was found to be, although not quite cured, very much relieved. He had no pain or spasmodic movements, and, in spite of his present contracted wryneck, he expresses himself as feeling that his condition is vastly better than it was before the operation. There was a condition of tonic wryneck in place of spasmodic wryneck. We can only mention the case reported by Mr. Edmund Owen,* we have not space to quote his thoroughly practical hints as to the details of his operation. The patient was a widow of mature age suffering from spasmodic contraction of the left sterno-mastoid muscle, by which the head was jerked down towards the left shoulder, the face being turned to the right side, and the chin thrown violently upwards. It had begun three years before, and was getting steadily worse. Five months after being operated upon the patient was again examined, and looked considerably younger than before the operation, having lost her distressed aspect, and having put on weight. The exhausting movements of the head had left her from the time of the neurectomy, though, to use her own words, "there is still a little something wrong; but it is improving." She held her head straight, and had perfect control over it. Mr. Pearce Gould's first case was that of a lady age 28, who had troublesome spasm of the left sterno-mastoid. It was so severe and constant as to prevent the patient from moving in society, and at night it was some time before the patient could get to sleep.

This was as long ago as September 10th, 1885, and Mr. Gould then exposed the spinal accessory nerve, intending to stretch it and excise a considerable portion. In stretching it from the central end it gave way, and he pulled out a long, slender nerve from the jugular foramen, and excised four inches of it. She appeared to have been completely cured at once, and a year later Mr. Gould was informed by Dr. Troutbeck, her medical adviser, that she was "quite well, except for occasional fatigue in the neck—no jerks." Mr. Gould has on two subsequent occasions intentionally removed the central end of the spinal accessory nerve in the same way for spastic torticollis. He remarks that the operation is quite a simple one, the delicate roots of the nerve rupture and a long tapering filament is drawn from the spinal canal.

It will be seen that the result depends on the more or less complete excision of the nerves to the muscles affected, and that when all these nerves can be operated upon the result is excellent. All depends on the nature of the case, for in some the operation consists simply in removing the spinal accessory, but in others it is a very difficult matter, first, to ascertain which muscles are affected, and then to get at some of the deep-lying nerves. What to aim at is, apparently, simple enough, and probably more full and careful investigation of the actions of the deep muscles of the neck will enable one to diagnose with greater certainty wherein the fault lies in any case, and to operate accordingly.

* *Lancet*, June 18th, 1892. † *New York Med. Journ.*, Mar. 5th, 1892.

* *Lancet*, June 18th, 1892.

